# Toxicity of amyloid beta 1-40 and 1-42 on SH-SY5Y cell line

**DOI:** 10.1186/2193-1801-4-S1-P19

**Published:** 2015-06-12

**Authors:** Jekaterina Krishtal, Olga Bragina, Kristel Metsla, Peep Palumaa, Vello Tõugu

**Affiliations:** Tallinn University of Technology, Estonia

**Keywords:** amyloid beta, toxicity, SH-SY5Y

Objectives: Amyloid beta plaques are primary hallmark of Alzheimer’s disease, which is characterized by specific neurodegeneration. Amyloid beta peptide – the main plaque component- was shown to be neurotoxic in animal models, primary neuronal cultures and immortalized cell lines. However, the results are often controversial and there is no good human cell line model for evaluation of the toxicity of amyloid peptides. Here we studied the effect of amyloid beta 1-40 and 1-42 on undifferentiated and differentiated human neuroblastoma cell line SH-SY5Y. Results: Undifferentiated cell culture was too diverse and unstable to reveal a toxic effect of amyloid beta peptides quantitatively. Differentiated cells established more neuron-like phenotype and were more identical and stable in culture suggesting potential susceptibility to amyloid beta as a neurotoxic agent. Amyloid peptides are prone to form different aggregates with diverse toxic properties, in current study, monomeric amyloid beta 1-40 and 1-42 were applied to the cells. Viability test WST-1 and propidium iodide (PI) uptake tests showed that undifferentiated cells are not susceptible to amyloid beta, however, differentiated cells showed reduced viability and increased PI uptake in case of amyloid beta 1-42, but not in case of amyloid beta 1-40. Conclusions: Current study revealed that amyloid beta has no remarkably toxic effect on undifferentiated SH-SY5Y cell line whereas viability of the neuron-like differentiated cell culture is significantly decreased by the amyloid beta 1-42 peptide that is known to form spontaneously toxic aggregates.

